# Successful Long-Term Preservation of Rat Sperm by Freeze-Drying

**DOI:** 10.1371/journal.pone.0035043

**Published:** 2012-04-09

**Authors:** Takehito Kaneko, Tadao Serikawa

**Affiliations:** Institute of Laboratory Animals, Graduate School of Medicine, Kyoto University, Kyoto, Japan; Clermont-Ferrand Univ., France

## Abstract

**Background:**

Freeze-drying sperm has been developed as a new preservation method where liquid nitrogen is no longer necessary. An advantage of freeze-drying sperm is that it can be stored at 4°C and transported at room temperature. Although the successful freeze-drying of sperm has been reported in a number of animals, the possibility of long-term preservation using this method has not yet been studied.

**Methodology/Principal Findings:**

Offspring were obtained from oocytes fertilized with rat epididymal sperm freeze-dried using a solution containing 10 mM Tris and 1 mM EDTA adjusted to pH 8.0. Tolerance of testicular sperm to freeze-drying was increased by pre-treatment with diamide. Offspring with normal fertility were obtained from oocytes fertilized with freeze-dried epididymal sperm stored at 4°C for 5 years.

**Conclusions and Significance:**

Sperm with –SS– cross-linking in the thiol-disulfide of their protamine were highly tolerant to freeze-drying, and the fertility of freeze-dried sperm was maintained for 5 years without deterioration. This is the first report to demonstrate the successful freeze-drying of sperm using a new and simple method for long-term preservation.

## Introduction

The rat is an important animal model that has been used to help understand the etiology of many human diseases [1], [2]. Presently, genetically engineered rat strains, not only transgenic [3], [4] but also knockout [5], are being produced. Furthermore, new technologies to generate knockout rats have been developed using microinjection of zinc-finger nucleases [6], [7] and transcription activator-like effector nucleases [8]. Sperm preservation is becoming an indispensable tool for preserving these rat strains as future genetic resources [9]. The cryopreservation of rat sperm has already been reported, and offspring were obtained from oocytes fertilized with cryopreserved sperm using *in vitro* fertilization (IVF) [10]. Although production of offspring using IVF is efficient, a considerable degree of technical skill is required to minimize damage to sperm motility due to environmental changes such as centrifugation [11], pH, viscosity, osmotic stress [12], [13], and the process of freezing and thawing [14]. Moreover, current IVF protocols are time-consuming with 5 hours incubation required for capacitation and 10 hours for penetration of sperm into oocytes *in vitro* [10], [15].

Freeze-drying sperm is expected to become a new preservation method because the use of liquid nitrogen is not required. An advantage of freeze-drying sperm is that it can be stored at 4°C [16]–[18] and stored and transported for short periods at room temperature without the use of liquid nitrogen and/or dry ice as cooling agents [18]. Attempts to freeze-drying sperm from several species of mammals have been reported [19]–[23]. A solution containing 10 mM Tris and 1 mM EDTA adjusted pH to 8.0 (TE buffer) has been used for freeze-drying mouse [24] and rat sperm [25], [26]. This buffer protects sperm DNA from physical damage by the freeze-drying process and activity of endogenous nuclease during storage [27], [28]. We have already obtained offspring from mouse and rat oocytes fertilized with sperm stored at 4°C for 1 year after freeze-drying using TE buffer [24], [26]. However, evaluation of long-term preservation exceeding 1 year of freeze-dried sperm is indispensable in the application of this new preservation method for bio-banking. We report here the condition of sperm, tolerance to freeze-drying, and the normality of freeze-dried rat sperm stored at 4°C for 5 years by evaluation of sperm fertility and analysis of DNA fragmentation.

## Results

Although no offspring were obtained from oocytes fertilized with freeze-dried testicular sperm, 8% of oocytes developed into offspring when fertilized with testicular sperm treated with diamide before freeze-drying. These were not significantly different from epididymal sperm (Table 1). Development of oocytes fertilized with freeze-dried rat sperm stored at 4°C for 5 years is shown in Table 2. Of the fertilized oocytes transferred into the oviducts of surrogate females, 20% implanted, and 11% developed into normal offspring. Two pairs of offspring were selected at random and their fertility was evaluated by mating at sexual maturity. All individuals derived from freeze-dried sperm stored at 4°C for 5 years showed normal fertility (Table 3).

**Table 1:** Development of oocytes fertilized with rat epididymal or testicular sperm after freeze-drying.^a^

Sperm collected from	Diamide treatment	No. of oocytes transferred	No. (%) of oocytes implanted^b^	No. (%) of offspring^b^
Epididymis	−	54	15 (28)^a^	6 (11)
Testis	−	36	0 (0)^b^	0 (0)
Testis	+	50	4 (8)^b^	4 (8)

a Freeze-dried ampoules were stored for less than 1 month.

b Percentages calculated from the number of oocytes transferred.

Within columns, percentages with different letters were significantly different (*P*<0.05) by analysis of χ^2^ tests with Yates correction for continuity.

**Table 2:** Development of oocytes fertilized with freeze-dried rat sperm stored at 4°C for 5 years.

No. of oocytes transferred	No. (%) of oocytes implanted^a^	No. (%) of offspring^a^
92	18 (20)	10 (11)

a Percentages calculated from the number of oocytes transferred.

**Table 3:** Fertility of individuals derived from oocytes fertilized with freeze-dried sperm stored at 4°C for 5 years.

Pairs	No. of offspring	No. of males	No. of females
A	16	11	5
B	13	7	6

**Figure 1:**
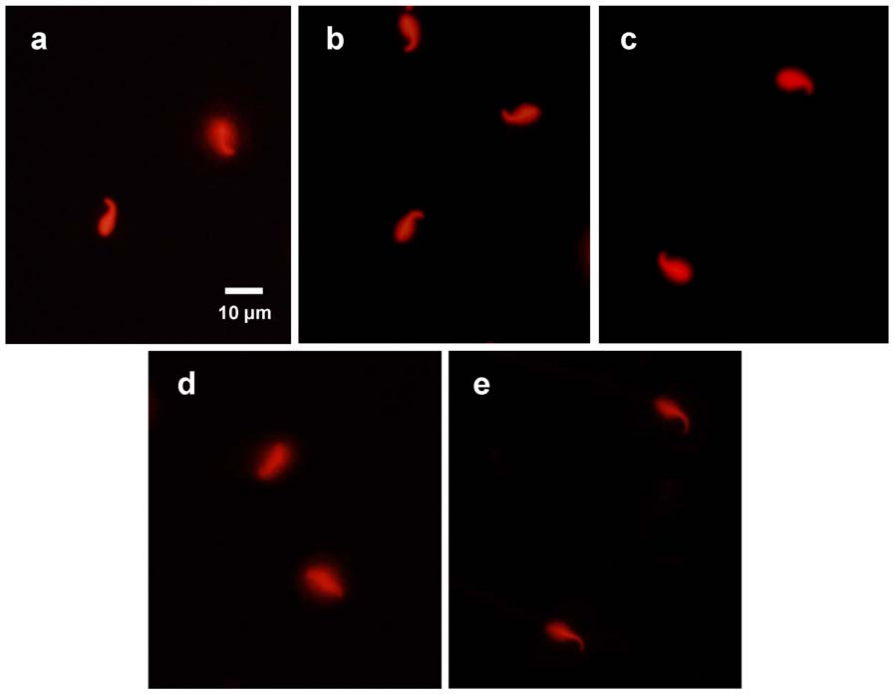
DNA fragmentation analyses of sperm. (a) Sperm with normal DNA (left) or fragmented DNA (right). Freeze-dried epididymal sperm stored at 4°C for 1 week (b) and 5 years (c). Testicular sperm freeze-dried (d) and freeze-dried after treatment with diamide (e) (original magnification 200×).

DNA fragmentation analyses of sperm are shown in Figure 1. Sperm with fragmented DNA showed a large and spotty halo indicating chromatin dispersion. Normal DNA with a small and compact halo of chromatin dispersion was shown in both freeze-dried sperm stored at 4°C for 1 week and 5 years. This would indicate that integrity of freeze-dried sperm has been maintained during the freeze-drying process and subsequent storage at 4°C for 5 years. Although the DNA of testicular sperm was fragmented after freeze-drying, sperm with normal DNA were obtained by pre-treatment with diamide prior to initiating the freeze-drying process.

## Discussion

We demonstrated that normal offspring can be obtained from oocytes fertilized with freeze-dried rat sperm stored at 4°C for 5 years (Table 2); these offspring demonstrated normal fertility (Table 3). Although we had already obtained offspring from oocytes fertilized with freeze-dried sperm stored at 4°C for 1 year [26], this study demonstrated that there was no decrease in the developmental ability of oocytes fertilized with freeze-dried sperm stored at 4°C for 5 years. Our study is the first to demonstrate such a prolonged and successful preservation of freeze-dried sperm, in any species, compared with previous reports.

The TE buffer used in our study is able to maintain the fertility of freeze-dried sperm without deterioration over the long-term. It is possible to use it as a simple preservation solution for the freeze-drying of both mouse [24] and rat sperm [25], [26]. Sperm preservation at 4°C by freeze-drying using TE buffer is simpler and more economical compared with preservation at −20°C, −80°C or in liquid nitrogen. Furthermore, freeze-dried sperm can be stored for up to 3 months at room temperature (24°C) with fertility conserved [18]. Freeze-drying sperm results in lower costs, as specialized equipment and a constant supply of liquid nitrogen is not required. Valuable samples can be temporarily stored at room temperature even in the event of a power failure, interruption to the liquid nitrogen supply or other emergencies by disaster such as earthquakes and typhoons. Short-term preservation of freeze-dried rat and mouse sperm at room temperature may also lead to easier transportation oversea and increased protection of valuable strains.

Although no offspring were obtained from oocytes fertilized with freeze-dried testicular sperm, the tolerance of testicular sperm to freeze-drying was increased by pre-treating with diamide (Table 1). These results demonstrate that the protection of sperm DNA is important for maintaining the fertility of freeze-dried sperm. We suggest that the chromatin of testicular sperm is extremely damaged by the physical stress of freeze-drying because the thiol-disulfide status of sperm nuclei was –SH [29]. Sperm with –SS– cross-linking, such as cauda epididymal sperm, remain fertile after the freeze-drying process [29], resulting in normal offspring obtained from oocytes fertilized with sperm stored at 4°C for 1 year after freeze-drying [24], [26]. Furthermore, our results show that no major DNA fragmentation was detected in freeze-dried sperm stored at 4°C for 5 years and offspring with normal fertility were obtained from oocytes. Diamide is an agent that oxidizes sperm protein –SH to –SS– [30]. Testicular sperm with –SS– cross-linking, when treated with diamide, were tolerant of damage from freeze-drying (Figure 1). However, chromatin of sperm with –SS– cross-linking was fragmented when a preservation solution with low pH [31], or lacking a chelating agent [24] was used for freeze-drying. The preservation solution for freeze-drying was modified by adding a small amount of EDTA and using a slightly higher pH (8.0) to protect sperm DNA from the activity of endogenous nuclease during storage [24]. The TE buffer developed in a previous study was found to strongly support the fertility of sperm during the freeze-drying process and subsequent long-term preservation at 4°C.

The number of live offspring produced from oocytes fertilized with freeze-dried rat sperm [26] was lower than that in mice [24]. However, major DNA fragmentation of freeze-dried rat sperm stored at 4°C for 1 year [26] and 5 years was not observed in this study. We previously reported that rat and mouse oocytes were considerably sensitive to the culture environment after fertilization [26], ICSI [32], [33], with differences observed between strains [33]. Further technical improvements to ICSI and embryo culture may lead to significantly improved developmental ability of oocytes fertilized with freeze-dried sperm.

In summary, long-term preservation of freeze-dried rat sperm at 4°C was achieved successfully, and offspring with normal fertility were generated from oocytes fertilized with the sperm. Sperm with –SS– cross-linking in their protamine such as cauda epididymal sperm and testicular sperm treated with diamide should be used because fertility is maintained during freeze-drying and subsequent long-term preservation at 4°C. We strongly believe that the freeze-drying process provides a safe and economical preservation of valuable rat strains, and provides us with a new method of sperm preservation for bio-banking.

## Materials and Methods

### Animals

All animal care and procedures performed in this study conformed to the Guidelines for Animal Experiments of Kyoto University, and were approved by the Animal Research Committee of Kyoto University.

Crlj∶WI rats were purchased from Charles River Japan Inc. (Yokohama, Japan). Male rats older than 11 weeks were used as sperm donors. Female rats used as oocyte donors were 4-weeks-old. All animals were maintained in an air-conditioned (temperature 24±2°C, humidity 50±10%) and light-controlled room (lights on from 07:00 to 19:00).

### Media

The TE buffer (10 mM Tris, 1 mM EDTA, pH 8.0; Applied Biosystems/Ambion, Austin, TX, USA) [24]–[26] was used for freeze-drying of sperm. The medium used for manipulation, including oocyte collection, oocyte handling and intracytoplasmic sperm injection (ICSI), was a rat 1-cell embryo culture medium [34], modified with 22 mM Hepes-Na and 20 mM sodium bicarbonate (H-mR1ECM).

### Sperm freeze-drying

Freeze-drying of sperm was carried out using the procedure described previously by Kaneko and his colleagues [18], [24]–[26], [29], [31]. Briefly, two cauda epididymides collected from the male were removed from blood and adipose tissue. A dense mass of sperm squeezed out of the cauda epididymides was gently places at the bottom of a 1.5 ml microcentrifuge tube in 1 ml TE buffer. The tube with sperm was left for 10 min at room temperature to allow the sperm to disperse into the solution. Testicular sperm collected from the fragments of seminiferous tubules were left untreated, or 5 mM diamide in 1 ml of TE buffer was added for 1 h in a culture dish. The supernatant (800 µl) was collected and 100 µl aliquots of both sperm suspension were transferred into a long-necked glass ampoule for freeze-drying (651506, Wheaton Science Products, Millville, NJ, USA). Ampoules were plunged into liquid nitrogen for 20 sec, and were then connected to the manifold of a freeze-drying machine (Freeze-drying systems 77530, Labconco Co., Kansas City, MO, USA). The sperm suspension was dried for 4 h at a pressure of less than 0.095 hPa. All ampoules were flame-sealed and preserved at 4°C.

### ICSI

Females were induced to superovulate by an intraperitoneal injection of 150 IU/kg pregnant mare serum gonadotropin (PMSG; Teikokuzoki Co., Tokyo, Japan) followed by an injection of 75 IU/kg human chorionic gonadotropin (hCG; Teikokuzoki Co.) 48 h later. Cumulus-oocyte complexes were collected from oviducts 14–16 h after hCG injection, and oocytes were freed from cumulus cells by treatment with 0.1% hyaluronidase in H-mR1ECM for 5 min. Oocytes were rinsed in fresh H-mR1ECM and kept at 37°C before ICSI.

Freeze-dried sperm were rehydrated by adding 100 µl of sterile distilled water. Rehydrated sperm were diluted in TE buffer, and 300 µl of sperm suspension was sonicated, to separate the sperm head from tail, for 1 sec using 30% power output of an ultrasonic homogenizer (VP-050, TAITEC Co., Ltd., Saitama, Japan).

A small volume (1–2 µl) of the sperm suspension was mixed thoroughly with a 10 µl droplet of H-mR1ECM containing 12% (w/v) polyvinylpyrrolidone (MW = 360,000; ICN Pharmaceuticals, Costa Mesa, CA, USA). A single sperm head with a normal shape was hung on the tip of an injection pipette. Sperm heads were then injected immediately into each oocyte. The oocytes after sperm injection were cultured in H-mR1ECM before transfer into surrogates.

### Oocyte transfer

Transfer of oocytes was carried out within 1 h after ICSI. The oocytes surviving after ICSI were transferred into the oviducts of surrogate females that were mated with vasectomized males the day before embryo transfer. Numbers of implantation sites and live offspring were assessed at 21 days of gestation. Live offspring derived from oocytes fertilized with freeze-dried sperm stored for 5 years were fostered to lactating females, and their fertility was evaluated by mating after maturation.

### Analysis of DNA fragmentation in freeze-dried sperm

DNA fragmentation was analyzed using Halomax (Halotech DNA SL, Madrid, Spain). Briefly, 4 µl of sperm suspension conducted in melting agarose was placed onto the marked wells of a glass slide. A glass coverslip was used to cover the sperm suspension, and the glass slide was cooled in a refrigerator for 5 min. The glass coverslip was gently removed, and then glass slide incubated in 10 ml of lysing solution at room temperature. After 5 min of incubation, the glass slide was washed with distilled water for 5 min. A glass slide was then dehydrated through a 70, 90 and 100% ethanol, and air-dried. Sperm stained with propidium iodide was used to analyze DNA fragmentation under an inverted microscope with a fluorescein filter (IX-71, Olympus Co., Tokyo, Japan).
